# Shifted intrinsic connectivity of central executive and salience network in borderline personality disorder

**DOI:** 10.3389/fnhum.2013.00727

**Published:** 2013-10-30

**Authors:** Anselm Doll, Christian Sorg, Andrei Manoliu, Andreas Wöller, Chun Meng, Hans Förstl, Claus Zimmer, Afra M. Wohlschläger, Valentin Riedl

**Affiliations:** ^1^Department of Psychiatry, Klinikum rechts der Isar, Technische Universität MünchenMunich, Germany; ^2^Department of Neuroradiology, Klinikum rechts der Isar, Technische Universität MünchenMunich, Germany; ^3^TUM-Neuroimaging Center, Technische Universität MünchenMunich, Germany; ^4^Munich Center for Neurosciences – Brain and Mind, Ludwig-Maximilians-Universität MünchenMartinsried, Germany; ^5^Department of Neurology, Klinikum rechts der Isar, Technische Universität MünchenMunich, Germany; ^6^Department of Nuclear Medicine, Klinikum rechts der Isar, Technische Universität MünchenMunich, Germany

**Keywords:** resting-state functional connectivity, brain networks, central executive network, default mode network, salience network, brain connectivity, large-scale networks, triple network hypothesis

## Abstract

Borderline personality disorder (BPD) is characterized by “stable instability” of emotions and behavior and their regulation. This emotional and behavioral instability corresponds with a neurocognitive triple network model of psychopathology, which suggests that aberrant emotional saliency and cognitive control is associated with aberrant interaction across three intrinsic connectivity networks [i.e., the salience network (SN), default mode network (DMN), and central executive network (CEN)]. The objective of the current study was to investigate whether and how such triple network intrinsic functional connectivity (iFC) is changed in patients with BPD. We acquired resting-state functional magnetic resonance imaging (rs-fMRI) data from 14 patients with BPD and 16 healthy controls. High-model order independent component analysis was used to extract spatiotemporal patterns of ongoing, coherent blood-oxygen-level-dependent signal fluctuations from rs-fMRI data. Main outcome measures were iFC within networks (intra-iFC) and between networks (i.e., network time course correlation inter-iFC). Aberrant intra-iFC was found in patients’ DMN, SN, and CEN, consistent with previous findings. While patients’ inter-iFC of the CEN was decreased, inter-iFC of the SN was increased. In particular, a balance index reflecting the relationship of CEN- and SN-inter-iFC across networks was strongly shifted from CEN to SN connectivity in patients. Results provide first preliminary evidence for aberrant triple network iFC in BPD. Our data suggest a shift of inter-network iFC from networks involved in cognitive control to those of emotion-related activity in BPD, potentially reflecting the persistent instability of emotion regulation in patients.

## INTRODUCTION

Borderline personality disorder (BPD) is characterized by “stable instability” (Schmideberg, 1959) of emotions, impulsivity, social relationships, and self-image. Additionally most patients suffer from chronic feelings of emptiness, complex dissociations, self-injury, and suicidal tendencies with a suicide rate of 10% (Oldham, 2006). BPD, which often co-occurs with other psychiatric disorders (about 85% of patients with BPD fulfill criteria for having at least one Axis I disorder; Lenzenweger et al., 2007), is common with a prevalence of more than 20% for psychiatric inpatients (Torgersen, 2005). Behavioral and emotional dysregulation is suggested as critical factors underlying this variety of symptoms (Leichsenring et al., 2011). We suggest that the stability of fluctuating symptoms across time and different situations might be related to consistent and profound functional alterations in the patient’s brain intrinsic functional architecture, particularly in brain regions involved in behavior/emotion regulation.

Previous functional neuroimaging studies revealed context specific patterns of altered brain activity in BPD patients during emotion- or self-related tasks. For example, negative emotional pictures or fearful/angry faces evoke stronger activity in the extrastriate, posterior cingulate, and frontal cortices, as well as weaker activity in the amygdala (Minzenberg et al., 2007; Koenigsberg et al., 2009a; Niedtfeld et al., 2010; Hazlett et al., 2012). In healthy subjects, self-distancing of negative pictures activates parietal regions overlapping with the so-called default mode network (DMN) including the medial prefrontal, medial and lateral parietal cortex (Koenigsberg et al., 2009b). Patients with BPD, however, fail to activate the DMN but show increased activity in the amygdala. On the contrary, memories of unresolved life events activate regions of the DMN in addition to amygdala, insula, and occipital cortices in patients (Beblo et al., 2006). Overall, emotional and self-related context increasingly activates an aberrant distributed pattern of brain regions including the DMN, insula, amygdala, and occipital cortices in BPD patients.

The measure of intrinsic functional connectivity (iFC), i.e., coherence of ongoing blood-oxygenation-level-dependent (BOLD) signal fluctuations in resting-state functional magnetic resonance imaging (rs-fMRI) data, is a surrogate for organized intrinsic brain activity (Fox and Raichle, 2007). At a large-scale level, coherent BOLD activity across remote brain areas forms consistent intrinsic connectivity networks (ICNs) in humans (Damoiseaux et al., 2006). Importantly, ICNs show strong spatial correspondence in independent analyses of resting-state and task-related activity patterns (Smith et al., 2009; Laird et al., 2011), suggesting that certain intrinsically coupled functional networks are also systematically engaged during cognition and behavior. Moreover, direct evidence exists that ongoing activity in ICNs serves as a scaffold for patterns of evoked neuronal activity (Keller et al., 2011), supporting the idea that the intrinsic architecture maintains and updates the brain’s repertoire of functional responses.

A recently proposed neurocognitive framework identified ICNs related to self-, emotion-, and cognitive control processing as neurocognitive “core” networks to study higher cognitive function and dysfunction (Menon and Uddin, 2010; Menon, 2011). In more detail, the anterior and posterior DMN (a/pDMN) covering the medial prefrontal cortex (mPFC), posterior cingulate cortex (PCC), and precuneus consistently activate during self-related and social cognitive functions (Buckner et al., 2008; Andrews-Hanna et al., 2010). The salience network (SN) covers anterior and posterior parts of the insula (AI, PI) and the anterior cingulate cortex (ACC) is critically involved in emotions, pain, and interoception (Seeley et al., 2007; Taylor et al., 2009; Legrain et al., 2011). Finally, left and right lateralized fronto-parietal networks (central executive network, CEN) are robustly associated with cognitive and executive control processes during goal-directed behavior (Seeley et al., 2007; Dosenbach et al., 2008; Habas et al., 2009). The consistent involvement of these three networks does not exclude other areas or networks to be also relevant for these functions particularly in specific contexts. However, it seems that these networks critically contribute (like a “core”) to self-, emotion-, and cognitive control-related processes (Menon, 2011), which are impaired in patients with BPD.

Several studies reported aberrant iFC within and across these ICNs in various neuropsychiatric diseases such as major depression (MD) or schizophrenia (Greicius, 2008; Hamilton et al., 2011; Uddin et al., 2011; Manoliu et al., 2013a,b) indicating the large-scale brain impact of these diseases on basic intrinsic functional network architecture and associated functions (for review, see also Menon, 2011; Palaniyappan and Liddle, 2012; Hamilton et al., 2013). Due to both the persistent nature of BPD and its “stable instability” in emotion-, self-, and control-related functions, we suggest altered iFC among DMN, SN, and CEN in BPD. In the so far only previous study focusing on iFC in BPD, Wolf et al. (2011) found aberrant (i.e., increased and decreased) iFC within the DMN and CEN of patients with BPD; but this did not yield information about the SN and the intrinsic connectivity across networks. To test our hypothesis about aberrant iFC within and across SN, DMN, and CEN in BPD, we acquired rs-fMRI data from patients with BPD and matched healthy controls (HC). We applied data-driven, high-model-order independent component analysis (ICA) to the rs-fMRI data to extract ICNs of coherent ongoing BOLD activity (Calhoun et al., 2001; Allen et al., 2011). We then examined the relationship, i.e., iFC, within (intra-iFC) and between (inter-iFC) ICNs-of-interest and provide a new measure capturing the balance across these neurocognitive networks.

## MATERIALS AND METHODS

### SUBJECTS

Fourteen right-handed patients and 16 age-, sex-, and handedness-matched HC participated in the study after signing the informed consent form in accordance with the Human Research Committee guidelines of the Klinikum Rechts der Isar, Technische Universität München (**Table 1**). Patients were recruited from the Department of Psychiatry, Klinikum rechts der Isar, Technische Universität München. Controls were recruited by word-of-mouth advertising from the larger Munich area. Participants’ examination included medical history, psychometric assessments [i.e., Beck Depression Inventory (BDI; Beck et al., 1961), Hamilton Depression Scale (HDS; Hamilton, 1960), short version of the Borderline Symptom List (BSL; Bohus et al., 2001), and Global Assessment of Functioning (GAF) Scale (Endicott et al., 1976)] and a structured psychiatric interview for patients only [Structured Clinical Interview for DSM-IV Axis I Disorders (SCID-I; First et al., 1996b) and Structured Clinical Interview for DSM-IV Axis II Personality Disorders (SCID-II; First et al., 1996a), German version]. All participants were examined by their psychiatrists (Andreas Wöller, Christian Sorg), professionally trained for SCID-based interviews with an inter-rater reliability of more than 95%. Psychiatric diagnoses were based on Diagnostic and Statistical Manual of Mental Disorders-IV (DSM IV).

**Table 1:** Demographics and psychometric scores.

Parameter	Patients with BPD	HC
*n*	14	16
Age (year)	30.4	34.0
Sex, male/female	1/13	1/15
GAF	43.7 ± 9.1^*^	100 ± 0
HDS	17.1 ± 7.4^*^	0.5 ± 0.8
BDI	18.1 ± 15.4^*^	1.8 ± 2.7
BSL	51.0 ± 17.4^*^	10.9 ± 3.9

*Data are presented as mean ± SD. HC, healthy controls; BPD, borderline personality disorder; GAF, Global Assessment of Functioning, HDS, Hamilton Depression Scale; BDI, Beck Depression Inventory; BSL, Borderline Symptom List;*

* *p* < 0.05 (two-sample *t*-tests).

Patients with BPD constitute a heterogeneous group of patients, who vary in diagnostic subcategories (e.g., with/without feeling of emptiness or stress-related paranoid ideation), comorbidity (e.g., with/without MD or post-traumatic stress disorder, PTSD), and degree of medication (e.g., with/without neuroleptica; Skodol et al., 2002). We adopted selection criteria for a representative group of patients recommended by Skodol et al. (1999) based on a longitudinal examination of 240 patients with BPD. BPD was the primary diagnosis for all patients. We excluded patients with current psychosis, intoxication, or confusional states, with a history of schizophrenia, schizoaffective disorder or bipolar disorder but we allowed co-occurrence of Axis I disorders MD or PTSD and psychotropic medication (Skodol et al., 1999). Additional exclusion criteria were an age below 18 or above 60 years, pregnancy, neurological or internal systemic diseases, and general contraindications for MRI assessment. A detailed description of each patient’s current comorbidity and medication can be found in **Table 2**. All control subjects were free of any current or past neurological or psychiatric disorder or psychotropic medication.

**Table 2:** Detailed clinical characteristics of patients with BPD.

Patients	Medication	Current comorbidity	History of comorbidity
1	Quetiapine 50 mg, Fluoxetine 20 mg	PTSD	Substance abuse
2	Olanzapine 5 mg, Quetiapine 600 mg (prolong), Escitalopram 20 mg	Alcohol abuse	MDD
3	Escitalopram 20 mg, Zopiclone 7.5 mg	Bulimia nervosa	Recurrent MDD
4	Quetiapine 100 mg, Lamotrigine 12.5 mg	Substance abuse, Cannabis dependence	Recurrent MDD
5	Quetiapine 300 mg (prolong), Sertraline 150 mg, Aripiprazole 10 mg	Multiple personality disorders	None
6	None	None	None
7	Atomoxetine 50 mg, Fluoxetine 20 mg, Paliperidone 3 mg	MDD, ADHD, alcohol abuse	Anorexia nervosa, recurrent MDD
8	Fluoxetine 40 mg	MDD	Substance abuse
9	Fluoxetine 30 mg, Quetiapine 12.5 mg, Pregabalin 225 mg	Undifferentiated somatoform disorder, alcohol abuse	Alcohol abuse
10	Aripiprazole 20 mg, Venlafaxine 150 mg	Alcohol abuse	Alcohol abuse
11	Pregabalin 300 mg, Quetiapine 60 mg, Venlafaxine 225 mg	PTSD, undifferentiated somatoform disorder, alcohol dependence	Recurrent MDD
12	None	None	None
13	Sertraline 75 mg	PTSD, substance abuse	Recurrent MDD
14	Sertraline 50 mg	Cannabis abuse	Recurrent MDD

*BPD, borderline personality disorder; PTSD, post-traumatic stress disorder; MDD, major depressive disorder.*

All participants in this study underwent 10 min of rs-fMRI with the instruction to keep their eyes closed and not to fall asleep. We verified that subjects stayed awake by interrogating via intercom immediately after the rs-fMRI scan. Before and after scanning, a medical examination of patients validated their stable condition and investigated whether they had feelings of odd situations during the scanning. No patient dropped out during the scanning session.

### MRI DATA ACQUISITION

Magnetic resonance imaging was performed on a 3-T whole body MR scanner (Achieva, Philips, Netherlands) using an eight-channel phased-array head coil. For co-registration of functional data, T1-weighted anatomical data were obtained from each subject by using a magnetization-prepared rapid acquisition gradient echo sequence [time to echo (TE) = 4 ms, repetition time (TR) = 9 ms, time for inversion (TI) = 100 ms, flip angle = 5°, field of view (FoV) = 240 mm × 240 mm, matrix = 240 × 240, 170 slices, voxel size = 1 mm × 1 mm × 1 mm]. fMRI data were collected using a gradient echo planar imaging (EPI) sequence (TE = 35 ms, TR = 2000 ms, flip angle = 82°, FoV = 220 mm × 220 mm, matrix = 80 × 80, 32 slices, slice thickness = 4 mm, and 0 mm interslice gap; an fMRI run of 10 min results in 300 volumes).

### fMRI DATA ANALYSIS

#### Preprocessing

For each participant the first three functional scans of each fMRI-session were discarded due to magnetization effects. SPM5^1^ (Wellcome Department of Cognitive Neurology, London) was used for motion correction, spatial normalization into the stereotactic space of the Montreal Neurological Institute (MNI) with resampling of voxel size to 3 mm × 3 mm × 3 mm, and spatial smoothing by applying an 8 mm × 8 mm × 8 mm Gaussian kernel. None of the participants had to be excluded due to excessive head motion (linear shift <3 mm across run and on a frame-to-frame basis, rotation <1.5°). Two-sample *t*-tests between groups yielded no significant results regarding translational and rotational movements of any direction as well as voxel-wise signal-to-noise ratio of fMRI data calculated with DPARSFA toolbox^2^ (*p* < 0.05).

#### Independent component analysis of fMRI data

Following a recent approach (Manoliu et al., 2013b), we applied high-model-order ICA to the preprocessed data by using the Group ICA of fMRI Toolbox (GIFT)-toolbox^3^ (version 1.3h) with the infomax algorithm implemented in Matlab (Calhoun et al., 2001). Data were decomposed into 70 spatial independent components (ICs), correspondent with a recently suggested framework for high-model-order decomposition (Abou Elseoud et al., 2011; Allen et al., 2011). High-model-order ICA approaches yield ICs, which are in accordance with large-scale functional networks from low-order approaches but offer a more detailed and particularly robust decomposition of sub-networks (Damoiseaux et al., 2006; Kiviniemi et al., 2009; Smith et al., 2009). Before volumes were entered into ICA analysis, voxel-wise *z*-transformation on time course data *y_ijk_*(*t*) was applied by subtracting the mean 〈*y_ijk_*〉 and dividing by the standard deviation σ*_ijk_ {ŷ_ijk_(t) = [y_ijk_(t) - 〈y_ijk_〉]/σ_ijk_*}, *t* time, *i,j,k* directions in space; Sorg et al., 2007). The sensitivity of the multivariate ICA algorithm for correlation of variance between voxels, i.e., functional connectivity, was thereby rendered independent of the original BOLD signal magnitude across subjects. Data were concatenated and reduced by two-step principal component analysis (PCA), followed by IC estimation with the infomax algorithm. We subsequently ran 40 ICAs (ICASSO) to ensure stability of the estimated components (Himberg et al., 2004). This results in a set of average group components, which are then back reconstructed into single subject space employing a dual regression analysis (group ICA (GICA) back-reconstruction approach (GICA-3) in GIFT; Erhardt et al., 2011). Each thus reconstructed IC results in a spatial map of *z*-scores reflecting the within-network iFC (intra-iFC) of a voxel within this component and an associated time course of BOLD signal fluctuations representative for this IC. We then reintegrated the initially calculated scaling factor σ*_ijk_* into the data by voxel-wise multiplication in order to preserve each individual’s profile of variance magnitude while leaving the normalized time course component unchanged.

#### Network selection

As previously described (Manoliu et al., 2013b), we ran a multiple spatial regression with a previously established baseline set of functionally relevant ICNs as regressors of interest (Allen et al., 2011) to automatically identify DMN, SN, and CEN in our dataset. From this publication, we selected the posterior (IC 53) and anterior (IC 25) DMN (a/pDMN), left and right lateralized fronto-parietal networks (ICs 34 and 60) reflecting left and right CEN, and an insular network (IC 55) reflecting the SN. The template for the insular network revealed a second component covering PI and bilateral amygdala and hippocampus [which we called posterior SN (pSN) in contrast to the anterior SN (aSN); see also Seeley et al., 2007; Taylor et al., 2009; Legrain et al., 2011]. Due to the importance of insular structures in BPD we also selected this component for further analyses.

#### Statistical analysis

To evaluate the spatial consistency of ICNs (intra-iFC), we calculated voxel-wise one-sample *t*-tests on participants’ reconstructed spatial maps using SPM5 for each ICN and group (*p* < 0.05, corrected for false discovery rate, FDR). We then examined group differences of intra-iFC. The individual *z*-maps were entered into voxel-wise two-sample *t*-tests and a conjunction map of the one-sample *t*-test image (*p* < 0.001 uncorrected) was applied as a mask to the analysis. In order to control for antipsychotic medication we added chlorpromazine (CPZ)-equivalent doses (Woods, 2003) as covariate-of-no-interest in all imaging analyses. The resulting SPMs were thresholded at *p* < 0.001 (voxel level) and *p* < 0.05 [corrected for family wise error (FWE) at cluster level].

In order to investigate group effects of inter-iFC *between* ICNs, we extracted each subject’s IC-timecourse of a/pDMN, l/r CEN, and a/pSN, calculated pairwise Pearson’s correlation coefficients between the time course of all ICNs for each subject, transformed the correlation matrix into *z*-values via Fisher *r*-to-*z*-transformation and tested differences between the two groups (two-sample *t*-tests with CPZ as covariate-of-no-interest, *p* < 0.05, Bonferroni-corrected for 15 pairwise correlations).

#### CEN/SN-inter-iFC index

Finally, we calculated the ratio (*r*) of overall inter-iFC for SN and CEN within the intrinsic functional architecture of DMN, SN, and CEN for each group controlling for effects of antipsychotic medication (two-sample *t*-test, *p* < 0.05): *r* = inter-iFC_sum_(CEN)/inter-iFC_sum_(SN). Here, the inter-iFC_sum_ reflects the inter-network connectivity of CEN and SN, and is calculated as the summarized absolute *z*-values of each network from the between ICN analysis. This integrated score is motivated by the idea that both SN and CEN interact with the DMN and among each other during emotion regulation, and that they are involved in cognitive control processes (task-positive networks; Seeley et al., 2007) with stronger representation of motivational/emotional aspects by the SN and of attention-related aspects by the CEN (Dosenbach et al., 2008; Menon, 2011; Hamilton et al., 2013).

## RESULTS

Psychometric assessment revealed significant differences between patients and controls for GAF (two-sample *t*-test, *t* = 17.3, *p* < 0.05), HDS (*t* = -7.1, *p* < 0.05), BDI (*t* = -3.1, *p* < 0.05), and BSL (*t* = -5.8, *p* < 0.05) between the two groups (**Table 1**).

### INTRA-iFC

**FIGURE 1:**
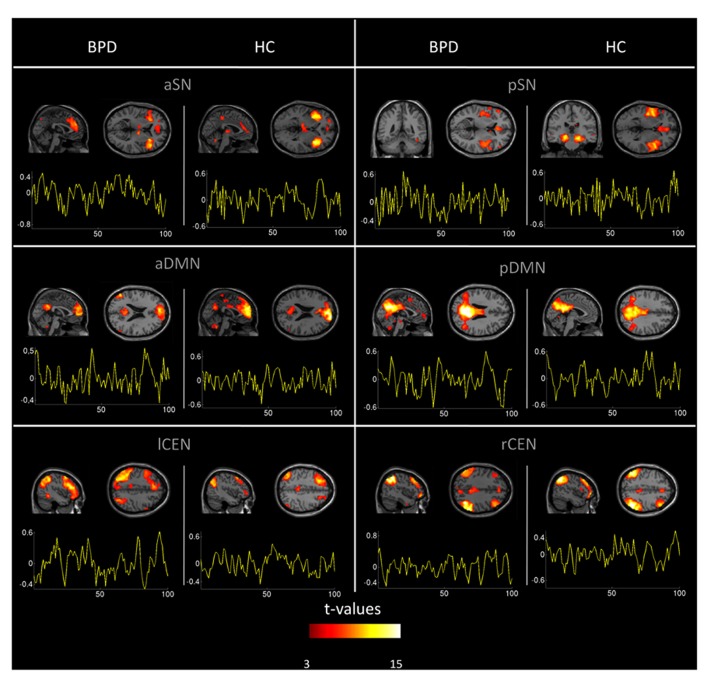
**Spatial maps and time courses of default mode, salience, and central executive network (DMN, SN, CEN) in healthy controls and patients.** Spatial statistical parametric maps (SPM, one-sample *t*-tests controlled for medication) and associated time courses of intrinsic networks in healthy controls (HC) and patients with borderline personality disorder (BPD). Maps and time courses are derived from independent component analysis of resting-state fMRI of subjects. SPMs are thresholded at *p* < 0.05 FDR-corrected and superimposed on a single subject high resolution T1 image. Color coding (red > yellow) represents *t*-values ranging from 3 to 25. The *x*-axis of signal time courses reflects number of fMRI scans; the *y*-axis represents normalized signal amplitude. First to third row: anterior and posterior (a/p) DMN, anterior and posterior SN, left and right (l/r) CEN.

Automated component selection, which was based on spatial templates representing subsystems of the DMN, SN, and CEN (see Figure 4 in Allen et al., 2011 for spatial templates), revealed six IC of interest from high-model-order analysis of fMRI data for each individual. The SN was represented in an anterior and posterior insular network (a/pSN), the DMN in an a/pDMN, and the CEN in left and right (l/r) CEN. Selected components were spatially consistent across groups and matched previous results of SN, DMN, and CEN (Allen et al., 2011; see **Figure 1** and **Table 3** for detailed description of intra-iFC within selected ICNs, *p* < 0.05, FDR-corrected).

**Table 3:** Spatial intra-iFC maps of DMN, SN, and CEN in controls and patients.

Networks and brain regions	HC					BPD				
	Cluster size	*t*_max_	MNI			Cluster size	*t*_max_	MNI		
			*x*	*y*	*z*			*x*	*y*	*z*
**aDMN**
Superior frontal gyrus	2506	18.39	15	63	21	1460	14.39	-6	54	15
Anterior cingulate cortex		17.68	6	48	21		12.18	-3	60	30
Inferior frontal gyrus						63	6.24	-45	33	-12
Middle cingulate cortex	56	4.8	0	-3	30					
Posterior cingulate cortex, precuneus	195	6.78	0	-60	27	330	12.9	-3	-51	33
Angular gyrus						266	10.91	-51	-66	30
Precentral sulcus	54	6.5	-6	-36	66					
Cerebellum	155	10.11	12	-54	-42	43	5.12	6	-54	-42
Putamen						25	5.41	-21	6	12
Middle occipital gyrus						74	5.08	57	-63	24
**pDMN**
Posterior cingulate cortex	2749	17.13	3	-48	21	4122	26.56	12	-48	30
Precuneus							22.45	-9	-57	33
Angular gyrus	247	15.48	45	-51	27					
Anterior cingulate cortex						39	3.87	6	39	21
Middle temporal gyrus	34	5.69	60	0	-21	79	4.87	-57	3	-24
Hippocampus						40	6.71	24	-36	-3
Cerebellum						47	4.12	-3	-24	-21
Fusiform gyrus						42	3.62	36	-75	-3
**aSN**
Right anterior insula	882	18.58	39	18	-3	723	18.88	48	24	-3
Left anterior insula	696	12.84	-33	9	-6	631	12.78	-30	27	-6
Orbitofrontal gyrus						53	4.94	-30	51	-3
Anterior cingulate cortex	275	6.41	9	39	15	868	8.19	-9	48	18
Superior medial gyrus										
Middle cingulate cortex	81	6.39	-6	-36	45		9.32	9	24	33
Thalamus	138	7.46	-9	-21	6	14	4.86	-9	-9	9
Cerebellar vermis	53	6.33	9	-57	-30					
Middle frontal gyrus	37	5.02	33	51	12		12.78	-39	27	-6
Angular gyrus	150	6.31	48	-45	30					
**pSN**
Right posterior insula	1239	11.6	48	9	0	679	14.82	51	-3	-12
Left posterior insula	892	11.03	-45	-12	3	487	11.96	-51	0	-6
Hippocampus	989	13.07	-15	-30	-6					
Anterior cingulate cortex	298	7.03	0	36	9	111	8.32	0	36	9
Inferior frontal gyrus	85	5.37	-48	30	15	50	5.91	-54	33	3
Right Amygdala	31	4.02	24	-3	-15					
**lCEN**
Middle frontal gyrus	1229	13.95	-24	23	59	2580	14.51	-45	36	18
Superior frontal gyrus		10.09	-15	36	51					
Inferior frontal gyrus						277	10.05	48	36	21
Superior medial gyrus	181	7.25	0	63	0					
Middle orbital gyrus	47	5.44	-42	48	-3					
Middle cingulate cortex	388	8.86	0	-36	36					
Thalamus						62	5.95	-6	-15	12
Inferior parietal lobe	128	6.52	48	-60	27	2071	14.46	-27	-66	39
							13.49	-48	-42	48
Superior temporal gyrus						45	4.86	66	-15	6
Insula						66	4.27	42	0	6
Hippocampus						44	6.5	-15	-3	-21
Cerebellum	298	7.78	39	-75	-30	293	8.35	30	-66	-33
Superior occipital gyrus						255	8.13	33	-72	45
**rCEN**
Middle frontal gyrus	1271	11.93	39	18	54	757	10.64	39	21	42
Middle orbital gyrus	54	4.92	-39	48	-9	220	8.54	36	51	-12
Middle cingulate cortex	85	7.68	3	-39	39	155	7.51	6	-45	33
Middle temporal gyrus	46	5.91	69	-42	0					
Inferior parietal lobule	641	19.16	-51	-54	45	665	10.65	-48	-54	48
Angular gyrus	1047	19.2	42	-60	39	967	20.56	42	-63	42
Precuneus						133	7.65	6	-78	42
Cerebellum	210	9.39	-36	-66	-42	124	7	-30	-66	-36
Fusiform gyrus						83	4.57	30	-66	-9

*One-sample *t*-test (corrected for medication), *p* < 0.05 corrected for false discovery rate. HC, healthy controls; BPD, borderline personality disorder; aDMN, pDMN, anterior and posterior default mode network; aSN, pSN, anterior and posterior salience network; lCEN, rCEN, left and right central executive network. Coordinates are presented in MNI standard space.*

**FIGURE 2:**
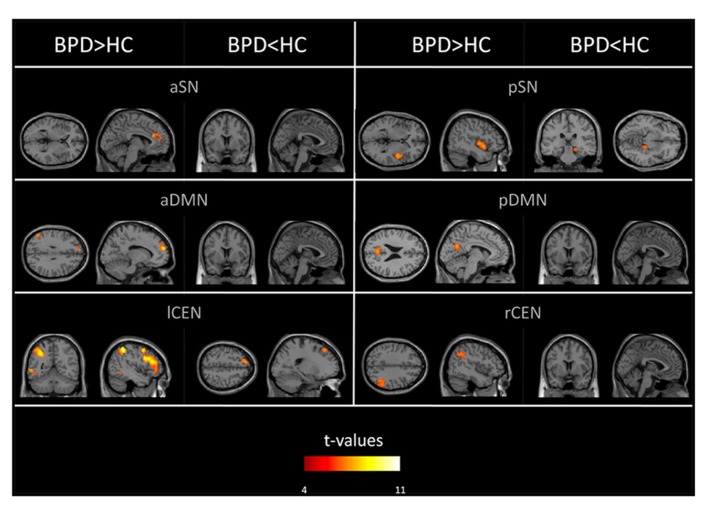
**Aberrant intrinsic functional connectivity within DMN, SN, and CEN (intra-iFC) of patients.** SPMs of group differences in intra-iFC for the DMN, SN, and CEN (voxel-wise two-sample *t*-tests) controlled for antipsychotic medication. SPMs are thresholded at *p* < 0.05, FWE-corrected at cluster level and superimposed on a single subject high resolution T1 image. Color coding (red > yellow) represents *t*-values ranging from 4 to 11.

Group comparisons of networks’ intra-iFC revealed regionally increased intra-iFC in each ICN of patients and decreased intra-iFC in only two ICNs (i.e., pSN, lCEN; *p* < 0.05 FWE-corrected cluster level and Bonferroni-corrected for six ICNs; **Figure 2**; **Table 4**). Increased intra-iFC in the BPD group covered various brain regions (*midline structures*: ACC, PCC, medial frontal gyrus; *parietal lobe*: bilateral SPL; *insula*: posterior part), decreased intra-iFC occurred in right hippocampus and left superior frontal gyrus.

**Table 4:** Group differences of intra-iFC maps for DMN, SN, and CEN.

Network with brain region	HC					BPD				
	Cluster size	*t*_max_	MNI			Cluster size	*t*_max_	MNI		
			*x*	*y*	*z*			*x*	*y*	*z*
**aDMN**
Left superior medial frontal gyrus						108	7.35	-9	57	24
Left superior frontal gyrus							3.92	-12	51	33
Intraparietal junction						55	3.42	-51	-63	33
**pDMN**
Left precuneus						207	6.67	-3	-63	24
**aSN**
Left superior medial gyrus						111	5.13	-9	48	18
Left anterior cingulate gyrus							4.15	-3	45	9
Right anterior cingulate gyrus							4.07	6	45	12
**pSN**
Right insular lobe						186	5.64	48	6	-6
Right hippocampus	38	4.45	21	-30	-12					
**lCEN**
Left precentral gyrus						639	8.12	-45	12	30
Left inferior frontal gyrus (pars triangularis)							7.77	-42	3021	
Left inferior parietal lobule						398	9.06	-45	-45	51
Left middle temporal gyrus						47	5.51	-57	-54	0
Left superior frontal gyrus	85	5.21	-15	36	51					
**rCEN**
Right angular gyrus						168	5.49	54	-48	30
Right inferior parietal lobule							4.54	45	-51	39

*Two-sample *t*-test (corrected for medication), *p* < 0.05 corrected for family wise error at cluster level and Bonferroni-corrected for six comparisons; green indicates increased intra-iFC in patients, red reduced intra-iFC. HC, healthy controls; BPD, borderline personality disorder; aDMN, pDMN, anterior and posterior default mode network; aSN, pSN, anterior and posterior salience network; lCEN, rCEN, left and right central executive network. Coordinates are presented in MNI standard space.*

### INTER-iFC

**FIGURE 3:**
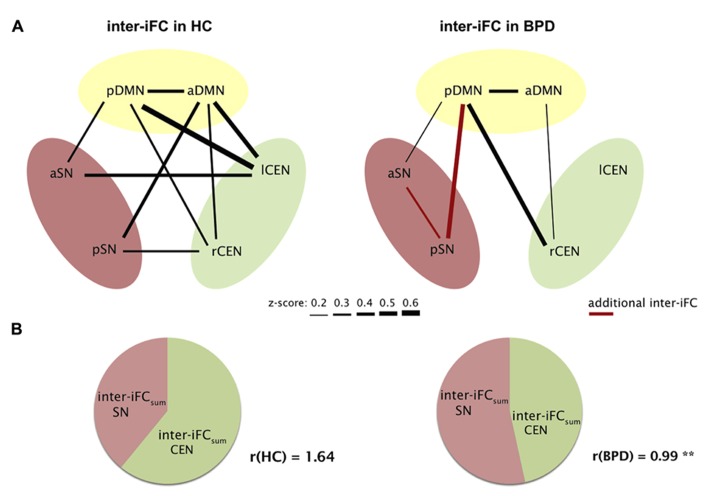
**Aberrant intrinsic functional connectivity between DMN, SN, and CEN (inter-iFC) of patients.** **(A)** Inter-iFC between two networks is based on Pearson’s correlation between network time courses. In healthy controls (HC), black lines indicate significant inter-iFC (one-sample *t*-tests, *p* < 0.05, Bonferroni-corrected for 15 correlations). Thickness of lines reflects absolute values of Fisher-*z*-normalized correlation coefficients. In patients with BPD, red lines indicate increased inter-iFC compared to healthy controls, while missing lines indicate significantly reduced and absent connections in BPD (two-sample *t*-tests, *p* < 0.05, Bonferroni-corrected). See also **Table 5** for correlation coefficients of significant inter-iFC. Results are controlled for antipsychotic medication. **(B)** For each subject, the ratio (*r*) of overall inter-iFC for CEN and SN within the intrinsic functional architecture of DMN, SN, and CEN was calculated by *r* = inter-iFC_sum_(CEN)/inter-iFC_sum_(SN), with inter-iFC_sum_ for CEN and SN, respectively, reflecting summarized absolute *z*-values of inter-iFC. We found significantly reduced *r* in patients (two-sample *t*-test, **p = 0.007).

To explore inter-iFC across DMN, SN, and CEN, we calculated the pairwise correlation between network time courses and tested significance of correlations and their potential group differences by using one- and two-sample *t*-tests controlling for effects of medication (CPZ covariate-of-no-interest). In HC, we found significant inter-iFC for 9 of 15 network pairs, while only four significant correlations occurred in BPD (*p* < 0.05, Bonferroni-corrected, black lines in **Figure 3A**; **Table 5**). The analysis of group differences revealed specific changes in the intrinsic functional architecture of patients (*p* < 0.05, Bonferroni-corrected for 15 connections; **Table 5**). More specifically, absent inter-network connectivity was found mainly for interactions concerning the CEN where four of six connections significantly decreased. Contrary to this overall decrease of iFC in patients, two additional intrinsic inter-network connections occurred in the patients group for the SN (red lines in **Figure 3A**).

**Table 5:** Inter-iFC between DMN, SN, and CEN.

Inter-iFC between ICNs	Healthy controls			BPD patients			Two-sample *t*-test (*p*)
	SEM	Mean	One-sample *t*-test (*p*)	SEM	Mean	One-sample *t*-test (*p*)	
aDMN–aSN	0.083	-0.089	0.300	0.112	0.144	0.220	0.177
aDMN–lCEN	0.059	0.472	0.000**	0.059	-0.036	0.550	0.000**
aDMN–pDMN	0.077	0.391	0.000**	0.086	0.417	0.000**	0.887
aDMN–pSN	0.067	0.361	0.000**	0.073	-0.063	0.406	0.000**
aDMN–rCEN	0.075	0.348	0.000**	0.086	0.262	0.010*	0.574
aSN–lCEN	0.089	-0.392	0.001**	0.079	0.014	0.860	0.003**
aSN–pSN	0.056	0.041	0.472	0.075	0.372	0.000**	0.009**
aSN–rCEN	0.084	-0.150	0.095	0.118	-0.017	0.889	0.323
lCEN–pDMN	0.064	0.563	0.000**	0.062	-0.112	0.095	0.000**
lCEN–pSN	0.090	-0.057	0.534	0.052	-0.035	0.507	0.783
lCEN–rCEN	0.112	0.236	0.053	0.097	0.056	0.574	0.220
pDMN–aSN	0.061	-0.347	0.000**	0.081	-0.236	0.012*	0.461
pDMN–pSN	0.082	-0.019	0.824	0.076	-0.446	0.000**	0.003**
pDMN–rCEN	0.083	0.354	0.001**	0.093	0.437	0.000**	0.672
rCEN–pSN	0.077	0.354	0.000**	0.089	-0.252	0.014*	0.000**

One-sample and two-sample *t*-tests (*p < 0.05 uncorrected, **p < 0.05, Bonferroni-corrected for 15 tests) including CPZ-equivalent doses as covariate-of-no-interest, for inter-iFC between intrinsic networks in healthy controls and patients with BPD (mean and standard error of Fisher *r*-to-*z*-transformed Pearson’s correlation coefficient among network time courses). aDMN, pDMN, anterior and posterior default mode network; aSN, pSN, anterior and posterior salience network; lCEN, rCEN, left and right central executive network.

Interestingly, in our correlation analysis of ICA-derived network time courses we found increased connectivity between the r/lCEN and a/pDMN in HC. This finding might be counterintuitive, since CEN and DMN are usually found anti-correlated (e.g., Fox et al., 2005). However, our findings for CEN and DMN sub-networks are perfectly in line with those of Allen et al. (2011), suggesting that such sub-networks are positively related among each other. This result might be explained by recent findings of Smith et al. (2012) based on a combination of high-model order spatial and temporal ICA; these authors demonstrated that the DMN can be subdivided into several functionally distinct sub-networks, each with its own characteristic patterns of correlations and anticorrelations with other intrinsic networks.

Finally, the observed global “shift” of inter-iFC among SN and CEN in patients was reflected by an altered CEN/SN-inter-iFC index *r* (**Figure 3B**). This ratio reflects the relative intrinsic impact of the CEN in comparison to the SN within the global intrinsic functional architecture of SN, CEN, and DMN. We found a significant difference between *r* (controls) = 1.64 ± 0.80 and *r* (BPD) = 0.99 ± 0.52 with *p* = 0.015 (two-sample *t*-test), potentially indicating a relative shift from cognitive control to emotion processing in patients with BPD (**Figure 3B**).

## DISCUSSION

The aim of this study was to investigate iFC among SN, DMN, and CEN in patients with BPD. This aim was motivated by previous findings demonstrating that interactions within and between these three networks contribute critically to behavior and emotion regulation; impaired emotion/behavior regulation, in turn, is suggested as an essential property of BPD. In a sample of 14 patients, we found aberrant intra-iFC in all three networks. While patients’ inter-iFC of the CEN was generally decreased, only inter-iFC of the SN was increased. In particular, a “balance” index reflecting the relationship of CEN- and SN-inter-iFC across networks was strongly shifted from CEN to SN connectivity in patients. This result provides first preliminary evidence for aberrant intrinsic connectivity among the DMN, SN, and CEN in BPD. Data suggest that patients’ impaired emotion/behavior regulation may rely on anomalous iFC among intrinsic networks that is centered on the SN.

### ABERRANT INTRA-iFC IN SALIENCE, DEFAULT MODE, AND CENTRAL EXECUTIVE NETWORK IN BPD

In patients, we found increased intra-iFC in the DMN, SN, and CEN with increases covering midline structures such as frontal and parietal cingulate cortices, prefrontal cortices (PFC), parietal lobes, and insular regions (**Figure 2**; **Table 4**). Decreased intra-iFC was found in right hippocampi and in the left dorsolateral frontal cortex (**Figure 2**; **Table 4**). Identified group differences were not due to a disintegration of investigated networks in patients, since basic spatial maps of networks were both largely consistent across groups (**Figure 1**; **Table 3**) and in line with previous findings (Damoiseaux et al., 2006; Allen et al., 2011). Patients’ counter-intuitively increased and decreased intra-iFC in intrinsic networks particularly in one and the same network (such as lCEN) has been observed also in other neuropsychiatric disorders such as schizophrenia (Manoliu et al., 2013b) or Alzheimer’s disease (Zhou et al., 2010) and – in line with our findings – in BPD (for the DMN and CEN; Wolf et al., 2011); however, the functional significance of the direction of intra-iFC changes in brain disorders is still unclear (e.g., iFC decreases are suggested to reflect connectivity disruptions while iFC-increases might reflect compensatory processes; but also a loss of desynchronization and therefore system complexity may play a role; Zhou et al., 2010). Previous imaging studies, which explored the neural correlates of impaired self- or emotion-processing in BPD, revealed aberrant task-related activity in areas similar to those of aberrant intra-iFC we found (Minzenberg et al., 2007; King-Casas et al., 2008; Driessen et al., 2009; Koenigsberg et al., 2009a; Smoski et al., 2011; Holtmann et al., 2013). For example, patients with BPD, who had to engage with emotional stimuli, had aberrant levels of activity in ACC, dorsolateral PFC, and amygdala (Minzenberg et al., 2007; Koenigsberg et al., 2009a; Holtmann et al., 2013); the insula was found to be the key region distinguishing BPD patients from HC in a more complex setting of a gambling task (King-Casas et al., 2008); in healthy subjects, self-distancing of negative pictures activates parietal regions overlapping with DMN (Koenigsberg et al., 2009b), while patients with BPD fail to activate the DMN. Furthermore, so far limited literature of resting-state imaging data in BPD supports the spatially widespread pattern of functional changes in BPD. A study using ^18^F-fluorodeoxyglucose-positron emission tomography (FDG-PET) found aberrant brain metabolism in prefrontal and cuneal regions (Juengling et al., 2003). Importantly, the only rs-fMRI study in BPD reported altered intra-iFC of prefrontal, cuneal, and insular regions within the DMN and CEN (Wolf et al., 2011), in line with our results. Taken together, our result demonstrates regionally specific iFC changes within DMN, SN, and CEN, which fit spatially previous findings of aberrant activity during tasks involved in emotion- and self-related processing.

### ABERRANT INTER-iFC AMONG DMN, SN, AND CEN IN PATIENTS

In addition, we found altered inter-iFC among DMN, SN, and CEN in patients (**Figure 3**; **Table 5**). More specifically, we observed an overall decrease of inter-iFC (with only two significant exceptions); this decrease of inter-iFC concerned mainly the CEN while increases were only found in the SN (**Figure 3A**; **Table 5**). The “shift” from a rather evenly spread inter-iFC pattern among the three networks in HC (**Figure 3A**) to a SN-centered pattern in patients (**Figure 3A**) was further indicated by a strongly reduced CEN-/SN-inter-iFC index (**Figure 3B**). The strong impairment of coordinated activity among these networks appears to be in line with a previous EEG study that found strongly impaired gamma-band synchrony in the parietal lobes of BPD patients during a cognitive control task (Williams et al., 2006). The most prominent cognitive model of BPD suggests that patients have deficits in emotion regulation due to impaired interactions between (pre-)frontal and limbic areas (Skodol et al., 2002; Mauchnik and Schmahl, 2010; Malhi et al., 2013). This is supported by several above-mentioned task-fMRI studies of either emotion processing (Minzenberg et al., 2007; Koenigsberg et al., 2009a) or cognitive control (Driessen et al., 2009; Koenigsberg et al., 2009b; Lang et al., 2012). Since these prefrontal–limbic areas largely overlap with the DMN, CEN, and SN, our results suggest an integrative model of altered intrinsic connectivity between emotion- and cognitive control-relevant intrinsic networks in BPD, which may be related to prefrontal–limbic regulatory deficits. This model implicates that neither system nor brain region alone is responsible for the various and stable behavioral symptoms in BPD. Future studies combining rs-fMRI and task-fMRI are necessary to test explicitly the relationship between aberrant iFC and emotion-evoked activity in BPD.

### PARALLELS WITH OTHER NEUROPSYCHIATRIC DISORDERS

Our result of aberrant iFC among DMN, SN, and CEN is largely consistent with the more general triple network hypothesis of psychopathology (Menon, 2011). This hypothesis states that psychopathological symptoms are associated with specifically altered coordinated activity across SN, DMN, and CEN; particularly, aberrant SN control function of DMN and CEN might underlie specific mental dysfunctions (Palaniyappan and Liddle, 2012). For example patients with schizophrenia with and without psychotic symptoms demonstrate distinctive changes of intra- and inter-iFC in the insular SN that are associated with impaired DMN/CEN interactions and positive and negative symptoms of patients (Manoliu et al., 2013a,b); in depressive patients, rumination is associated with aberrant coordination of intrinsic SN, DMN, and CEN activity (Hamilton et al., 2011). Concerning BPD, our data suggest that impaired behavior/emotion regulation might be associated with SN-centered inter-iFC reorganization of triple network functional architecture; however, more explicit evidence for such specific link between network interaction changes and behavioral deficits in BPD is necessary (for more detailed discussion of this point see below “limitations”). Furthermore, in comparing among different disorders one has to pay attention to potential confounding effects of psychotropic medication, which might be used in both compared disorders, e.g., antipsychotics in BPD and schizophrenia. Based on these findings, three basic questions about the specificity of aberrant triple network iFC in BPD arise: how specific are iFC changes for distinct psychopathological symptoms such as emotional response style or impulsivity in BPD? Beyond symptoms, how specific are iFC changes for comparisons with other neuropsychiatric disorders? Beyond triple network, which further brain changes outside the triple network such as subcortical or neurochemical changes are critical for distinct symptoms or differences with other disorders? To disentangle such questions, future studies, which may include different psychiatric disorders and brain measures beyond iFC, are necessary.

### LIMITATIONS

First, although comparable with previous studies in BPD, the sample size of our study is small (*n* = 14; e.g., Koenigsberg et al., 2009a; Wolf et al., 2011; Lang et al., 2012). In general, a small sample size reduces the power of effects, and increases the likelihood of false positive results (Button et al., 2013). Therefore the presented results are preliminary and warrant further replication with higher sample sizes. Second, our patient sample is heterogeneous due to gender, comorbidity, and medication status. This heterogeneity is due to clinically based inclusion criteria, which provided a clinical representative patient sample (Skodol et al., 1999). On the one hand this heterogeneity together with small sample size precluded us to link brain changes with specific behavioral changes; in such groups, the distribution of symptom severity is too heterogeneous to allow for brain–behavior relationship analysis. On the other hand, our results are independent of specific BPD sub-groups, suggesting that observed changes of triple network iFC are a general feature of BPD. Nevertheless, studies in more homogeneous sub-groups of BPD might be helpful to specify aberrant network iFC due to BPD sub-groups. Third, patients of the study were therapeutically treated with psychotropic substances (**Table 2**). While we did control for antipsychotic medication, we did not control for antidepressant medication because no appropriate numerical procedure (comparable to CPZ conversion) is available for antidepressants. Previously, antidepressant effects on brain activity and functional connectivity have been discussed for the BOLD signal (Miller et al., 2001; Phillips et al., 2008; Heller et al., 2013). Although recent studies suggest that antidepressants normalize brain function (Anand et al., 2005; Fu et al., 2007; Heller et al., 2013), we cannot exclude antidepressant medication effects on our results. Future studies of non-medicated patients are necessary. Forth, some limitations concerning the use of ICA to identify ICNs have to be considered. Our selection of a model order 70 was empirical; although a model order of about 75 components seems to be an optimal choice (Abou-Elseoud et al., 2010), no clear computational or objective criterion for that number is available. Furthermore, the selection of ICNs of interest from ICA-derived components is intricate, particularly due to subjective bias; to account for this problem, we performed maximally controlled spatial regression analysis of all ICs on ICN templates as previously described (Manoliu et al., 2013b), which stem from a previous study using a very similar approach (Allen et al., 2011).

## CONCLUSION

The current study provides evidence for aberrant iFC within and across DMN, SN, and CEN in patients with BPD. Data suggest a “shift” of inter-network iFC from networks of cognitive control to those of emotion-related activity, potentially reflecting the persistent instability of emotion regulation in patients.

## Conflict of Interest Statement

The authors declare that the research was conducted in the absence of any commercial or financial relationships that could be construed as a potential conflict of interest.

## Footnotes

1

2

3
